# ULtimate MAGnetic characterization (ULMAG) at the ID12 beamline of ESRF: from element-specific properties to macroscopic functionalities

**DOI:** 10.1107/S1600577524011718

**Published:** 2025-02-03

**Authors:** Alex Aubert, Andrei Rogalev, Gabriel Gomez, Elvina Dilmieva, Johanna Lill, Benedikt Eggert, Konstantin Skokov, Fabrice Wilhelm, Heiko Wende, Katharina Ollefs, Oliver Gutfleisch

**Affiliations:** a Functional Materials, Material Science TU Darmstadt 64287Darmstadt Germany; b European Synchrotron Radiation Facility 38000Grenoble France; c Faculty of Physics and Center for Nanointegration Duisburg-Essen (CENIDE) University of Duisburg-Essen Duisburg47057 Germany; University of Essex, United Kingdom

**Keywords:** ULMAG, X-ray magnetic circular dichroism, XMCD, simultaneous measurements, magnetism, functional materials

## Abstract

We present a novel instrument designed for advanced magnetic study, installed at the ID12 beamline of the European Synchrotron Radiation Facility in Grenoble, France. This instrument offers the unique capability to simultaneously measure element-specific microscopic and macroscopic properties related to the magnetic, electronic and structural characteristics of materials. In addition to X-ray absorption, X-ray magnetic circular dichroism alongside X-ray diffraction patterns, the macroscopic magnetization, volume changes, caloric properties and electrical resistivity of magnetic materials could be measured strictly under the same experimental conditions as a function of both magnetic field (up to ±7 T) and temperature (ranging from 2.05 K to 325 K). To demonstrate the capability of this new instrument, we present two case studies highlighting its performance in investigating first-order magneto-structural phase transitions, namely in DyCo_2_ and FeRh alloys.

## Abstract 2

We present a novel instrument at ID12 (ESRF) capable of simultaneously measuring element-specific microscopic and macroscopic properties related to the magnetic, electronic and structural characteristics of functional materials.

## 1. Introduction

The discovery of X-ray resonant magnetic scattering in 1985 (Namikawa *et al.*, 1985 ▸), X-ray magnetic linear dichroism in 1986 (van der Laan *et al.*, 1986 ▸) and X-ray magnetic circular dichroism (XMCD) in 1987 (Schütz *et al.*, 1987 ▸) marked a significant breakthrough in magnetism research, allowing for the pursuit of objectives that were previously unattainable. XMCD spectroscopy, in particular, has become an essential tool in understanding the magnetism of complex materials due to its inherent specificity to elements and orbitals, as well as their ability to probe small sample volumes. The formulation of magneto–optical sum rules has further strengthened XMCD, offering a unique capability to quantitatively determine orbital and spin contributions to the total magnetic moment carried by the absorbing atom (Thole *et al.*, 1992 ▸; Carra *et al.*, 1993 ▸).

In the early 1990s, the emergence of third-generation synchrotron radiation facilities provided the scientific community with X-ray spectroscopy beamlines featuring enhanced flux and controllable polarization. As a result, polarization-dependent X-ray spectroscopy has become a standard tool in contemporary magnetism research over the last 25 years. This has led to a deeper understanding of the microscopic origins of the magnetic state of matter and significant technological advancements (Rogalev *et al.*, 2013 ▸; Le Guyader *et al.*, 2023 ▸).

However, remaining at the forefront of emerging areas in magnetism research requires the ability to correlate the element-specific properties measured with X-ray absorption spectroscopy (XAS) and XMCD with the macroscopic properties measured in laboratories. As an example, this is essential for multi-functional materials that exhibit a close interplay between their structural, magnetic and electronic components (Gutfleisch *et al.*, 2011 ▸; Gutfleisch *et al.*, 2016 ▸). However, these different subsystems are typically examined independently and in separate samples, environments or even laboratories. This fragmented approach makes it challenging to assemble these disparate pieces of the puzzle into a coherent picture. Consequently, it is not surprising that the mechanisms governing the coupling of, for example, magneto-structural transitions remain poorly understood (Skokov *et al.*, 2023 ▸).

Thus, there is a compelling need for the development of a novel experimental approach that integrates element-specific microscopic and macroscopic measurements conducted under the same conditions, such as temperature and magnetic field, all on a single sample. This approach has been tackled in our ULtimate MAGnetic characterization (ULMAG) project, leading to a dedicated endstation at the ID12 beamline at the European Synchrotron Radiation Facility (ESRF, France). Two setups have been developed in the framework of this project. In the first setup, we use the existing high-field XMCD endstation based on a 17 T solenoid and demonstrate the possibility to measure XAS and XMCD spectra simultaneously together with magnetization, temperature of the sample and strain (Aubert *et al.*, 2022 ▸). Remarkable possibilities enabled by our approach have been illustrated with the study of the field-induced first-order phase transition in FeRh (Aubert *et al.*, 2022 ▸).

**Figure 1:**
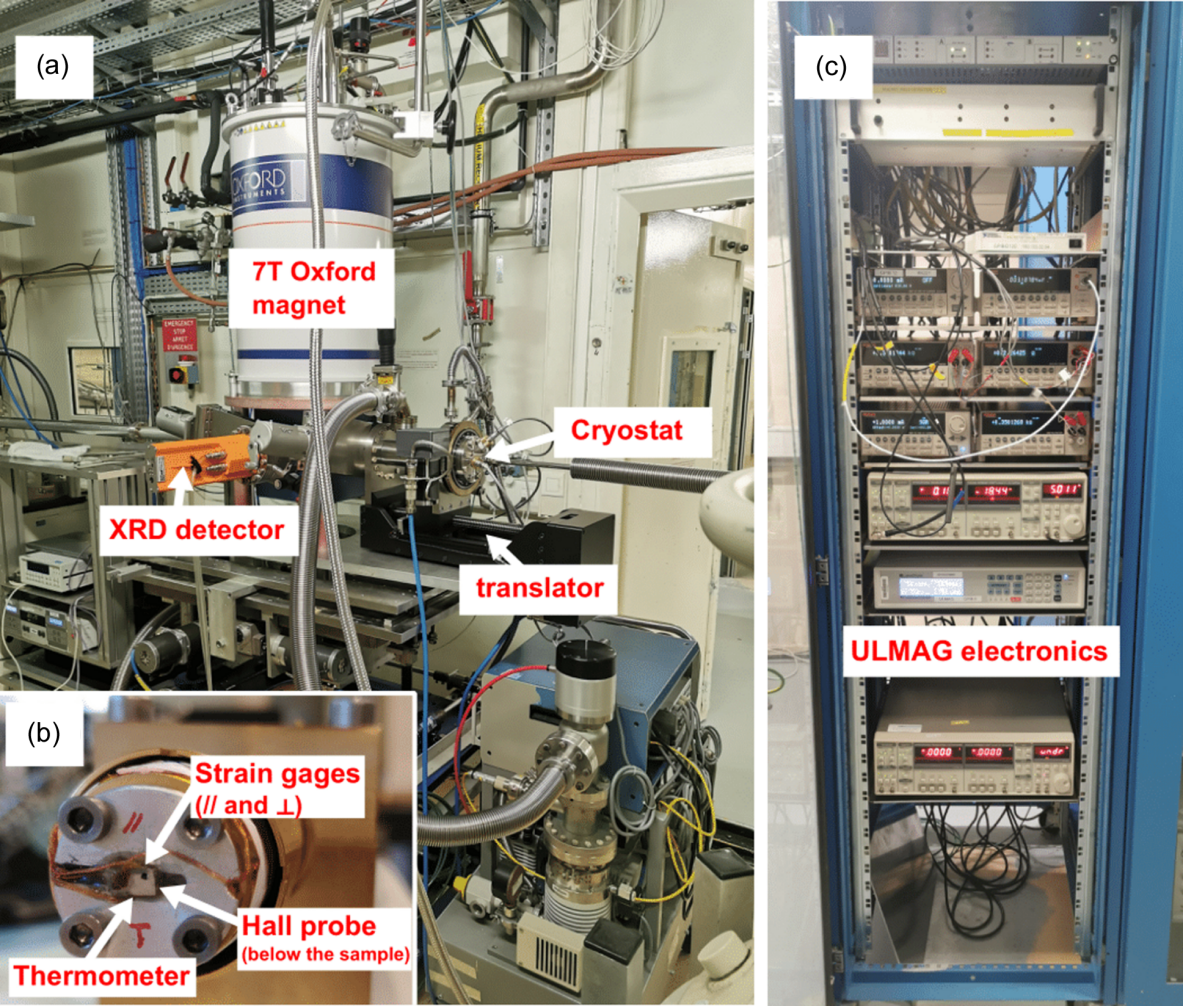
(*a*) Photograph of the experimental hutch at ID12 with the ULMAG setup, (*b*) a sample with sensors on the holder and (*c*) the electronics rack.

Here, we describe a new ULMAG setup that was recently installed and commissioned at the ESRF beamline ID12 (see Fig. 1 ▸). It is based on a dedicated 7 T split coil magnet which allows one to measure the XAS and XMCD spectra under strictly the same experimental conditions together with X-ray diffraction and various macroscopic properties (*i.e.* magnetization, sample temperature, volume change and resistivity). Following the brief description of the beamline and ULMAG endstation itself, we present several case studies (DyCo_2_ and FeRh) which exhibit magnetic phase transitions to demonstrate the capability of the new instrument.

## 2. The ESRF beamline ID12

The main scientific emphasis of beamline ID12 is the magnetic properties of materials explored with polarization-dependent X-ray spectroscopy in the tender and hard X-ray energy ranges (Rogalev & Wilhelm, 2015 ▸). What makes this beamline specific is that the polarization state of the X-ray beam can be fully controlled over a wide energy range from 2 keV to 15 keV. This covers the *K*-edges of elements from P to Br including all 3*d* transition metals, *L*_2,3_-edges of the 4*d*/5*d* transition metals and rare-earths, as well as the *M*_4,5_-edges of actinides.

The XMCD spectra are recorded using a total fluorescence yield detection scheme with subsequent correction for re-absorption effects, taking into account the chemical composition of the sample, the fluorescence background of the matrix and the solid angle of detection. An important asset of this detection mode, with its bulk sensitivity, is that it is equally suitable to perform experiments on thin films, buried layers and bulk samples.

Up to now, three experimental stations for XMCD experiments were available at the ID12 beamline. The first is dedicated for measurements of soft magnetic materials and based on Bruker electromagnet BE-15V. It includes a central hole with a diameter of 10 mm in the poles for incoming X-rays and generates a magnetic field up to 0.9 T. The temperature of the sample can be varied between 10 K and 600 K.

The second XMCD endstation is built around an 8 T superconducting solenoid and optimized for experiments under high pressure using diamond anvil cells (Wilhelm & Rogalev, 2024 ▸). The magnet features a 100 mm-diameter room-temperature bore and a true four-quadrant power supply offering fast sweep option, up to 4 T min^−1^. The adapted standard ESRF diamond anvil cell is mounted inside the main heat exchanger of the dedicated ‘amagnetic’ cryostat designed at the ESRF. The base temperature of this cryostat is 2.0 K, which corresponds to a measured temperature inside the diamond anvil cell of 2.7 K.

Finally, the high-field XMCD endstation is based on a high-vacuum (<10^−7^ mbar) superconducting solenoid magnet, producing a horizontal magnetic field as high as 17 T (Rogalev & Wilhelm, 2013 ▸). A dedicated helium continuous flow ‘amagnetic’ cryostat was developed at the ESRF. The temperature of the heat exchanger can be set in the range 2.05 K to 325 K with a stability of Δ*T*/*T* ≤ 10^−3^. This setup has been adapted for the first simultaneous measurements of XAS/XMCD together with macroscopic measurements [magnetization, temperature of the sample and strain (Aubert *et al.*, 2022 ▸)]. It served us as a prototype for a new fully dedicated endstation described below.

## 3. ULMAG endstation

A superconducting split-coil magnet (Spectromag 4000-8 manufactured by Oxford Instruments NanoScience) generating magnetic fields of up to ±7 T is the central part of the ULMAG experimental station. This is a wet system with 50 mm cold bore and 38 mm split. It is powered by a 20 V 120 A power supply enabling rapid ramping of magnetic fields at a rate of 1 T min^−1^.

The tail of the outer vacuum chamber of the magnet has five access ports. One is located at the bottom and is used for pumping the line and uses a CF63 flange. The two ports along the split feature CF100 flanges and can be used for mounting a 2D detector for X-ray diffraction or to introduce an optional cryostat with the sample to measure the powder diffraction pattern under field in transmission geometry. Eventually, an energy-dispersive X-ray detector based on a silicon drift detector with on-chip collimated active area of 80 mm^2^ and low-capacitance ASIC (CUBE) primary amplification devices can be installed at one of the ports. The two ports along the magnetic field direction are also equipped with CF100 flanges: one at the front, which is used for the incoming X-rays and XAS detector insert, and one at the back, which is used to introduce the cryostat with the sample using a 250 mm translation stage.

The cryostat is a shorter version of the helium continuous flow ‘amagnetic’ cryostat which was developed for the high-field XMCD setup at ID12. In addition, the ULMAG cryostat is equipped with 3 × 12-pin LEMO connectors to connect the sensors with the measurement devices.

**Figure 2:**
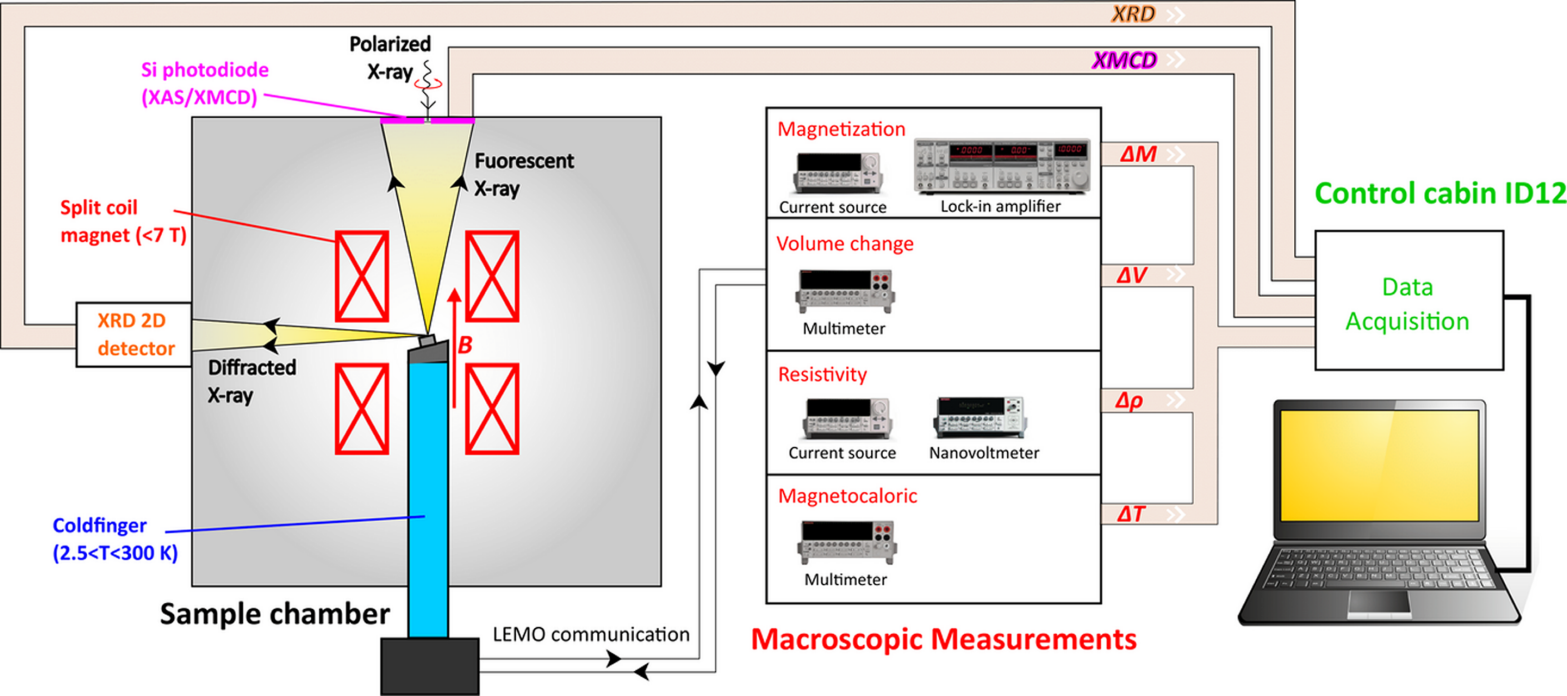
Schematic of the ULMAG setup implemented at beamline ID12 of the ESRF.

Fig. 2 ▸ illustrates a schematic of the ULMAG setup installed and developed at the ID12 beamline of the ESRF. The sample is glued on an aluminium sample holder, which is mechanically attached to a cold finger of the helium constant flow cryostat. The temperature of the sample can be precisely regulated within the range 2.05 K to 325 K while maintaining a high degree of stability, approximately 0.1%. Similarly to the high-field XMCD setup, X-ray fluorescence photons emitted by the sample are detected in back-scattering geometry with the silicon photodiode featuring an oblong hole, mounted directly on the liquid nitrogen shield of the superconducting magnet. To measure the macroscopic properties, various types of sensors must be affixed prior to the measurement, either by adhering them to the sample or the aluminium sample holder. All electronic devices are linked to the beamline control computer through a GPIB/LAN interface, seamlessly integrated into the SPEC control software of the ESRF. This allows us to perform the macroscopic measurements simultaneously with XMCD. The measurement of macroscopic properties does not affect the acquisition time, which is limited by the XMCD signal and depends on samples and edges to be measured.

The use of the ULMAG setup requires an accurate sample preparation prior to beam time, as various sensors must be attached to the samples and tested beforehand (Aubert *et al.*, 2022 ▸). A manual is available for users.

## 4. Measurement capabilities of the ULMAG setup at the ID12 beamline

### 4.1. X-ray diffraction

Among the novel capabilities of the ULMAG instrument is the possibility to perform X-ray diffraction measurements under the same conditions as XAS and/or XMCD spectra. The remarkable brightness of synchrotron X-rays ensures excellent counting statistics on the detector in a short timeframe, facilitating rapid collection of high signal-to-noise data that would be unattainable on a laboratory instrument. Additionally, the small source size at the ESRF-EBS enables easy focusing of the X-ray beam to a small spot size of a few micrometres, and in-line optics exploiting beryllium refractive lenses. Consequently, this reduces the minimum sample volume required for adequate data quality and offers the possibility to examine complex or heterogeneous materials with a few micrometres spatial resolution.

This functionality introduces the potential to monitor structural phase transitions and even structural distortions. By observing the behaviour of a particular Bragg reflection, alterations in lattice parameters, reduction of symmetry (manifested via splitting of a diffraction peak) and other related changes can be identified. Detection of a diffraction pattern is accomplished with a commercial Lambda 750k detector designed by X-Spectrum.

#### 4.1.1. Detector

The Lambda 750k is a 2D single-photon-counting X-ray detector based on Medipix3 technology. This is a large area detector with 1528 × 516 pixels and a pixel size of 55 µm. It is equipped with an Si sensor allowing a count rate capability up to 200000 counts pixel^−1^ s^−1^ and high detection efficiency in the photon energy range from 3.5 keV to 25 keV. This matches very well with the energy range available at ID12 beamline. To avoid air flow window scattering in experiments at these X-ray energies, the systems is operated under vacuum. Note that for diffraction experiments, X-rays photons with energies up to 40 keV can be used at ID12, by exploiting high-order harmonics of the undulators. To gain intensity of higher-order harmonics from a helical undulator one has to set it up in elliptical mode.

#### 4.1.2. Geometry

**Figure 3:**
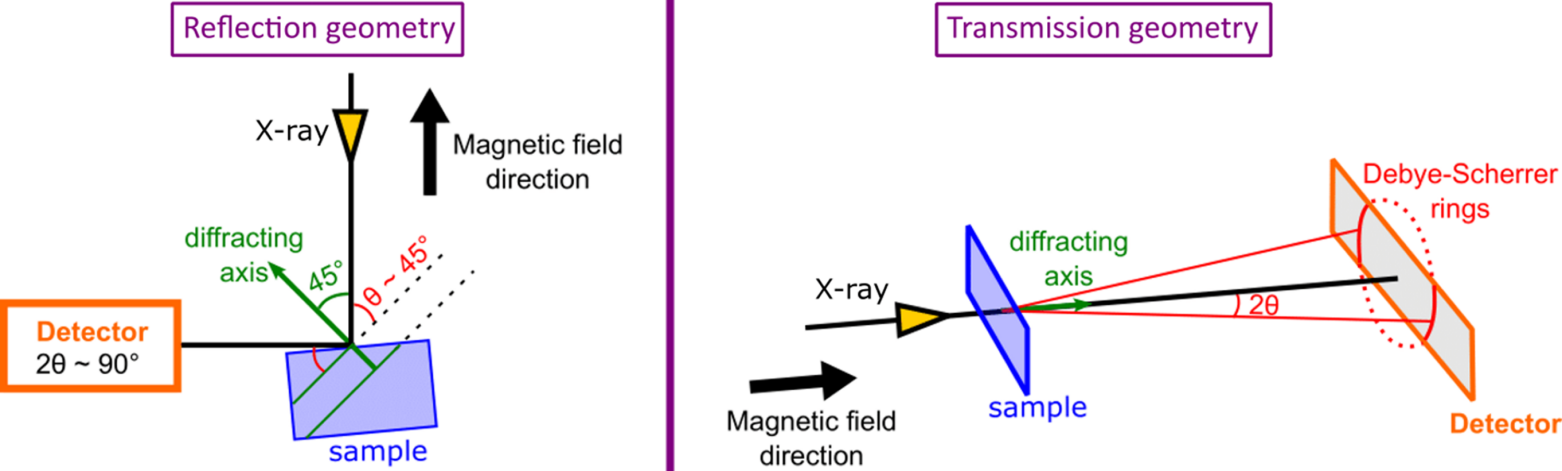
Geometry of XRD measurements in (left) reflection and (right) transmission mode.

**Figure 4:**
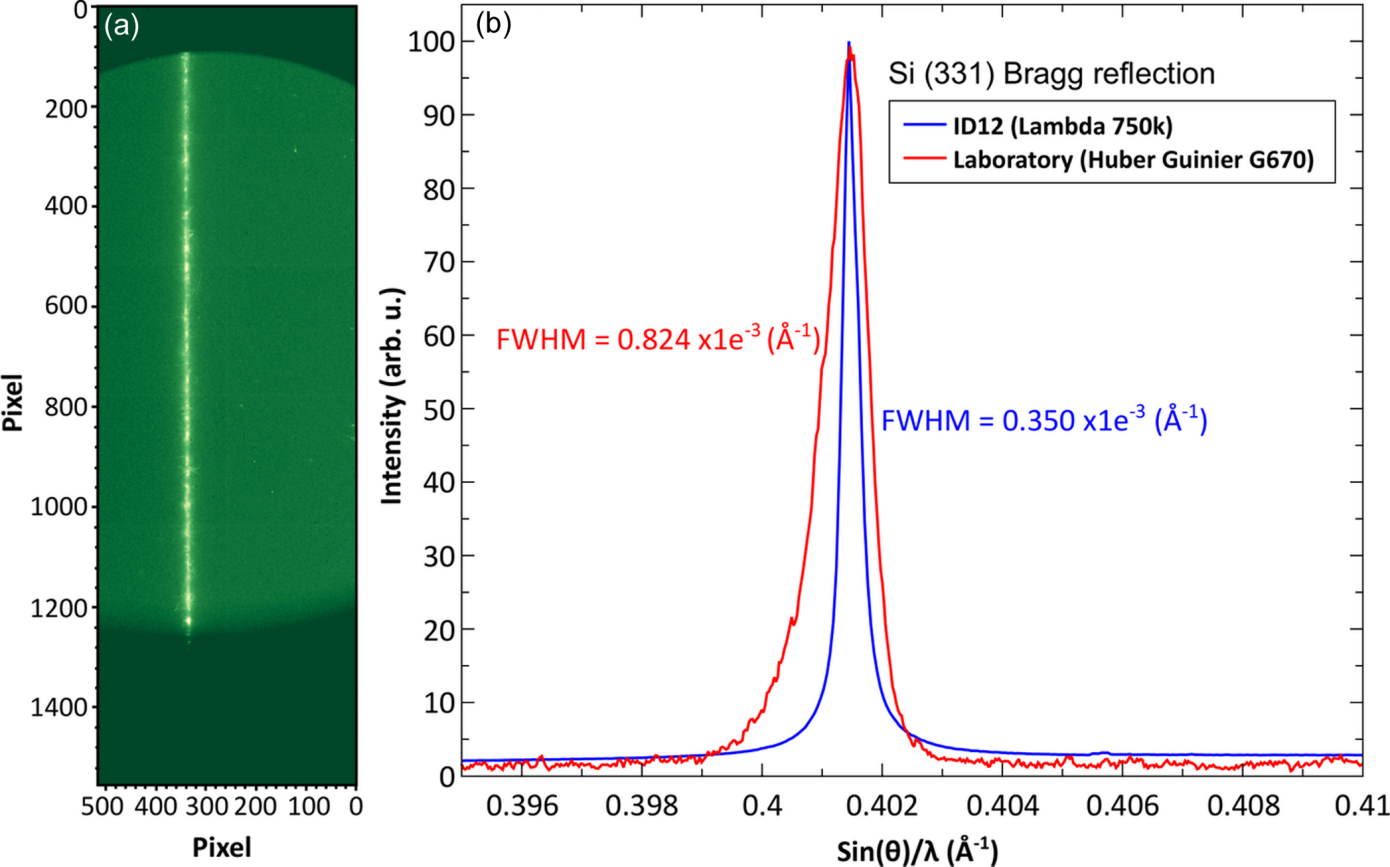
(*a*) Si reflection of the (331) peak as measured on the 2D detector at an energy of 7.018 keV and (*b*) after integrating the signal over various rows and comparison with laboratory measurements and their respective full width at half-maxima.

For bulk samples, the XRD detector is fixed at around 90° (reflection mode) from the incident beam, which is allowed by the geometry of the split-coil magnet (see Fig. 2 ▸). In this case, the diffracting planes of the samples would be orientated about 45° (±3°) from the incident beam and consequently from the applied magnetic field as shown in Fig. 3 ▸. Note that, in this configuration, only the vertically polarized component of the X-ray beam is diffracted. A typical powder diffraction pattern exhibits vertical lines on the 2D detector as illustrated in Fig. 4 ▸(*a*), showing 331 diffraction from Si powder using circularly polarized X-rays of 7.018 keV.

In addition, we also have the possibility to place the detector in transmission mode (see Fig. 3 ▸). In this case, the diffraction manifests as Debye rings.

In reflection mode, the detector is typically located at a fixed distance of ∼25 cm from the sample. At this specific distance, a resolution of ∼0.015° pixel^−1^ is achieved, providing an angular range of ∼7° around 90° in 2θ. Since the θ angle is fixed at around 45°, one has to tune the photon energy to obtain given diffraction peaks at the detector. The detector can be optionally mounted on a translation stage that adds the ability to change the distance between the detector and the sample, and therefore can increase the angular resolution by a factor of five but at the cost of the reduced angular acceptance of the detector.

A direct comparison of the instrumental resolution between a typical laboratory diffractometer (Guinier camera G670) with a primary beam monochromator using an Mo *K*α source in transmission geometry and our experimental setup at ID12 beamline is possible when the width of the diffraction peak is expressed in the momentum transfer space *q* = sin(θ)/λ. We compare in Fig. 4 ▸(*b*) the (331) diffraction peak of Si measured with 7.018 keV circularly polarized X-rays and on a laboratory diffractometer. Even though the Guinier G670 has a smaller step size (0.0025°) than in our current setup (0.015°), we have more than twice better angular resolution due to the excellent quality of the X-ray beam at ID12 beamline.

In transmission mode, the detector is positioned horizontally at a fixed distance of ∼15 cm from the sample. At this specific distance, an angular resolution of ∼0.02° pixel^−1^ is achieved, providing a full angular range of ∼30°. Similarly, the detector could be mounted on a translation stage, but again there is a trade-off between the desirable angular resolution and accessible angular acceptance of the detector.

**Figure 5:**
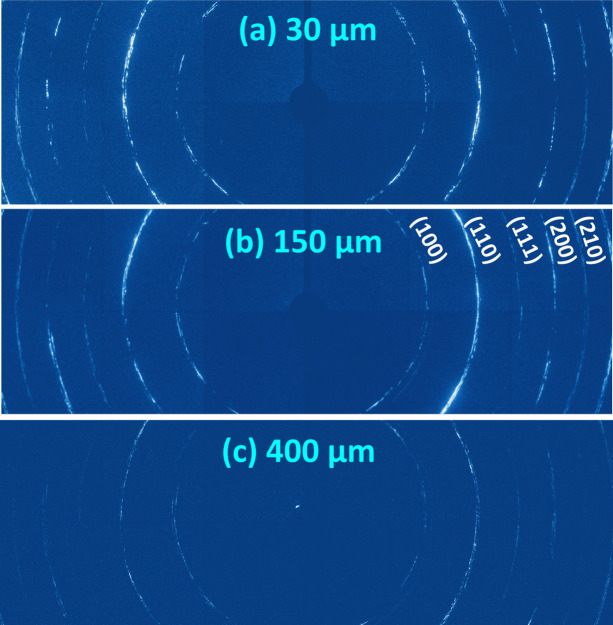
Diffraction rings of three FeRh samples of (*a*) 30 µm, (*b*) 150 µm and (*c*) 400 µm thicknesses, measured at 36 keV.

In this geometry, one has to bear in mind that the X-ray penetration depth in the sample is the limiting factor, *i.e* the thickness of the sample has to be optimized to reduce the attenuation of the beam. For example, X-ray diffraction patterns of an FeRh sample with thicknesses of 30 µm, 150 µm and 400 µm are shown in Fig. 5 ▸.

These have been measured with X-ray photons of 36 keV. To minimize the background contribution due to X-ray fluorescence from the sample, the energy threshold of the detector was set above the Rh *K* absorption edge (∼24 keV). For the 30 µm- and 150 µm-thick samples, well defined diffraction rings are observed in Fig. 5 ▸(*b*). Inhomogeneities of grain size and their orientation within the sample are reflected in the discontinuity and variable intensity along the diffraction rings. For the 400 µm-thick sample, much weaker rings are visible [Fig. 5 ▸(*c*)] and longer acquisition times would be necessary to obtain high-quality data.

### 4.2. Magnetization

We have previously shown that, under proper conditions, the magnetic stray field produced by the sample is proportional to the sample bulk magnetization (Aubert *et al.*, 2022 ▸). The stray field is measured using a Hall probe (HP) incorporated into the aluminium sample holder, with its active surface positioned as close as is feasible to the sample – almost in direct contact. The HP is stimulated by a high-precision AC current, and its reaction to the overall magnetic field (including the applied field and sample’s stray field) is recorded as a Hall voltage. The sensors are commercially available GaAs based HPs. In our setup, we use the HP AST 214 T from Asensor Technology AB, which has a resistance of approximately 500 Ω and a theoretical sensitivity of 0.2 V T^−1^ at 2 mA excitation current. It has the smallest geometry available for a cabled sensor, being approximately 2 mm wide and 0.5 mm thick, which make it easy to embed using epoxy in the aluminium sample holder.

**Figure 6:**
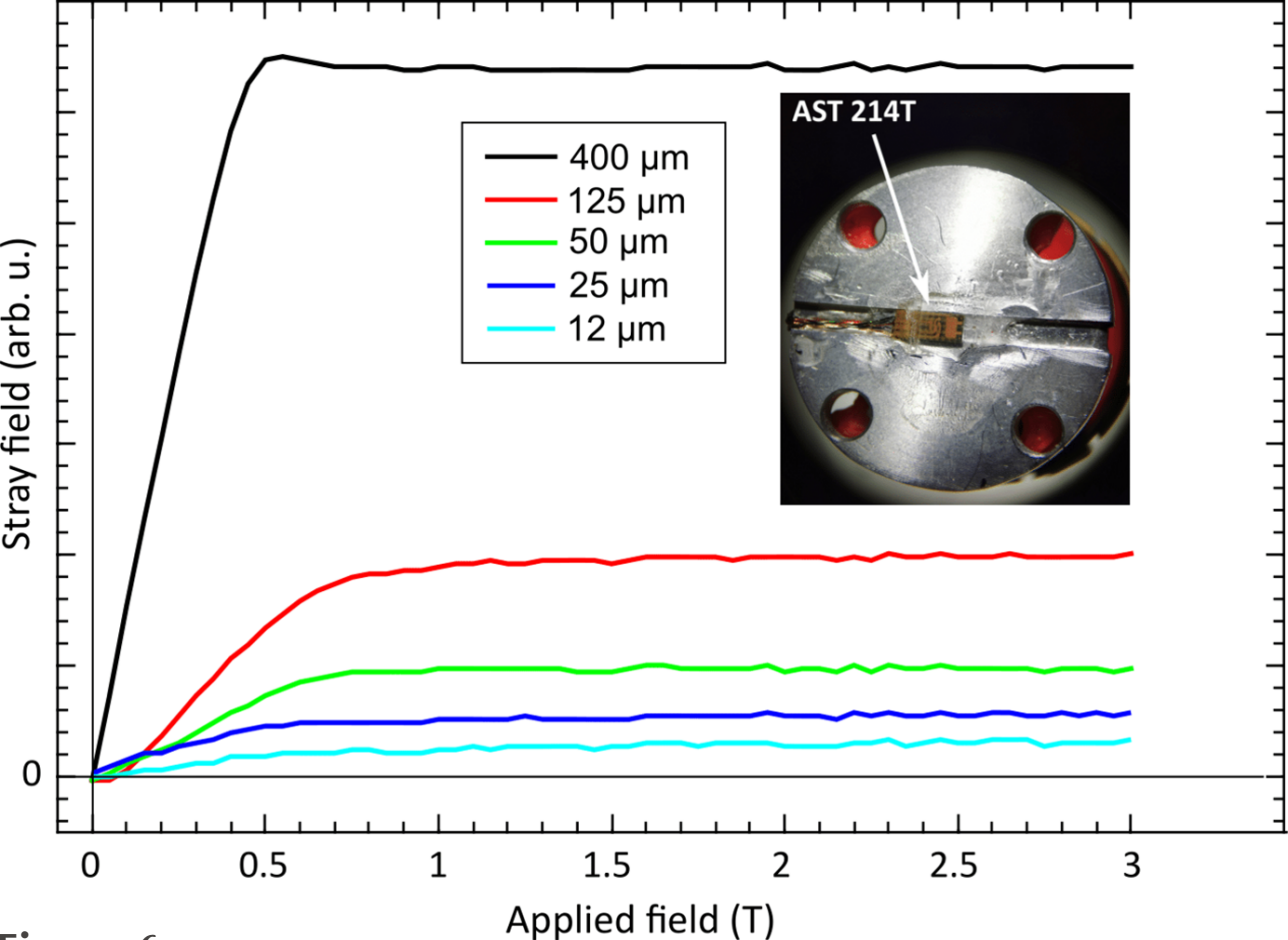
Stray field measured with HPs for different thicknesses of Ni disk of 6 mm diameter. The inset shows the embedded HP in the aluminium sample holder.

To evaluate the sensitivity limit of magnetization measurements with the HP, we measured the stray field of thick foils of Ni with a 5 mm × 5 mm area and varying thicknesses. The results for the five different samples are depicted in Fig. 6 ▸. The stray field can be detected for Ni samples as thin as 12 µm, corresponding to a moment of 0.15 mA m^2^. However, it is always desirable to compare the curves obtained by the HP with those from a standard vibrating sample magnetometer (VSM)/SQUID to ensure the measurement quality.

### 4.3. Magnetostriction

The relative length change of the sample is measured by means of two Vishay Micromeasurement strain gauges (model SK-06-031CF-350). The gauges are connected to a high-precision multimeter (Keithley 2002) used in the four-terminal sensing mode to measure their resistance. Two strain gauges (1.5 mm × 1.5 mm) are glued to the surface of the bulk sample using M-Bond glue from Vishay Micromeasurement. One gauge is used to measure the changes in length along the direction of the applied magnetic field (Δ*L*/*L*_∥_) and the second is used to measure the changes in the perpendicular direction (Δ*L*/*L*_⊥_), thus allowing us to calculate the volume change in the sample if isotropic.

The formula to calculate the change in length (in p.p.m.) is
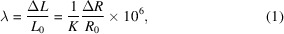
where *K* is a gauge factor (in our case it is in the range 1.9–2.1), *L*_0_ is the initial length of the sample and *R*_0_ is the initial resistance of the strain gauge.

### 4.4. Magnetocaloric properties

The magnetocaloric effect is measured by means of a Cernox resistance thermometer (model CX-1050-BC) glued directly on the surface of the sample. The resistance is measured with a high-precision multimeter (Keithley 2002) in the four-wire resistivity mode. Variation of the sample temperature (δ*T*/*T*) can be measured from cryogenics temperature (2 K) up to 400 K. However, the sample is not under adiabatic conditions.

### 4.5. DC resistivity

The resistivity of a sample can be measured with various devices depending on the conductivity of the sample. For high-precision resistivity with a conductive (*e.g.* metallic) sample, we use a nanovoltmeter (Keithley 2182) and a current source (Keithley 6221) paired together and use the ‘Delta mode’ option. In this case, the measured resistance can be as low as 10 nΩ. The sample should ideally be in the form of thin wires or bars with uniform cross-sectional areas and wire contact soldered at the edges for precise measurements. For insulating samples (resistivity > 100 MΩ), a Keithley 2002 multimeter model offers the possibility to accurately measure resistance up to 1 GΩ.

The resistivity is calculated using the following equation,
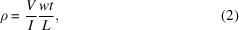
where *V* is the voltage, *I* is the current, *wt* is the cross-sectional area and *L* is the distance between electrodes (*i.e.* the length of the sample).

### 4.6. Complementary capabilities

The ULMAG instruments are versatile and can be straightforwardly complemented by other measurement tools that rely on using voltage and/or current. Techniques like AC resistivity (electrical transport), Hall transport *etc.* can be easily implemented.

## 5. Case studies

### 5.1. DyCo_2_

Laves phase compounds *RM*_2_ (*R* = heavy rare-earth, *M* transition metal) exhibit diverse magnetic phase transitions that could be used in magnetocaloric liquefaction of hydrogen (Liu *et al.*, 2022 ▸, 2023 ▸, 2024 ▸). In DyCo_2_, there is a metamagnetic transition from a paramagnetic to a ferrimagnetic state occurring around 140 K (Bloch & Lemaire, 1970 ▸; Inoue & Shimizu, 1982 ▸; Gratz & Markosyan, 2001 ▸; Bykov *et al.*, 2024 ▸). Here, the magnetic coupling between the heavy rare earth Dy and Co moments is antiparallel. It has been proposed that the order of the phase transition depends on various factors, including the molecular field provided by the *R* 4*f* magnetic moment, the itinerant Co 3*d* electron band and spin fluctuations (Hauser *et al.*, 2000 ▸). XMCD has been performed on this compound to assess how changes in the magnetic moment of the rare-earth and 3*d* sublattices (XMCD) that appear during magnetic phase transitions correlate with macroscopic changes in the net magnetization of the sample. This also allowed us to determine how changes in the lattice parameters measured by X-ray diffraction correlate with macroscopic changes in sample size (thermal expansion, magnetostriction).

**Figure 7:**
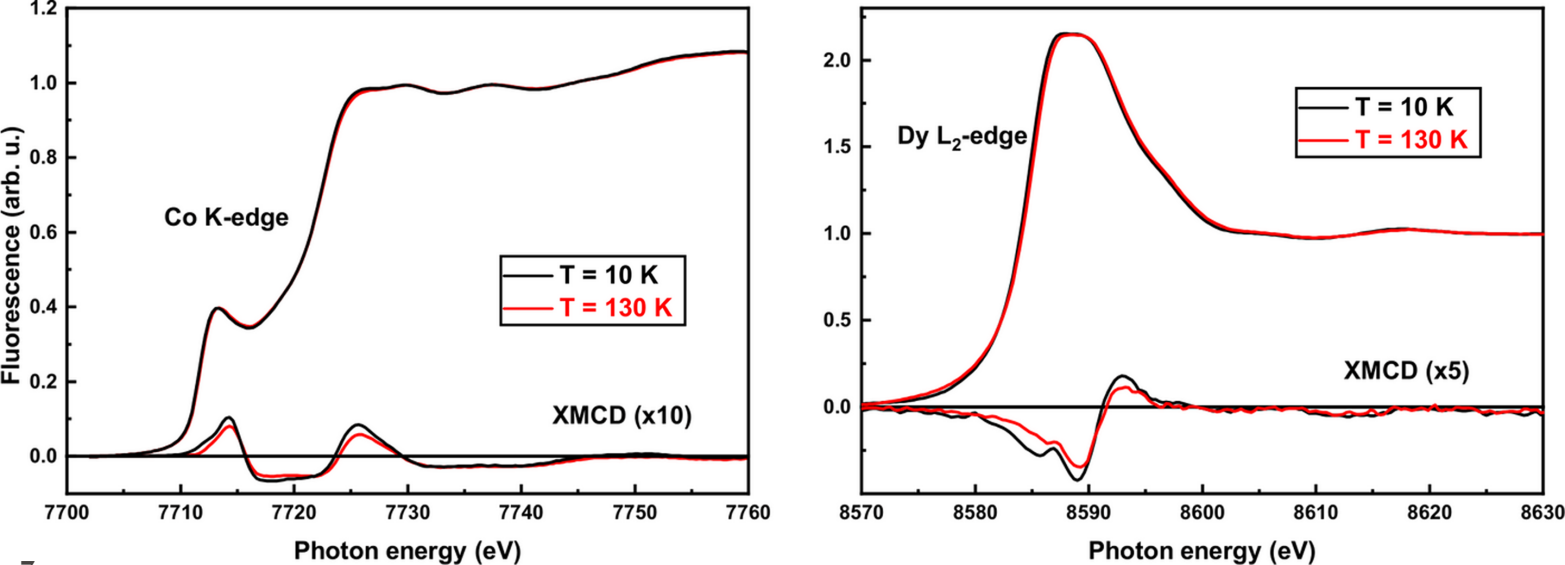
XANES (top curves) and XMCD (bottom curves) data of the DyCo_2_ sample at different edges and temperatures. The applied magnetic field is 6.5 T.

**Figure 8:**
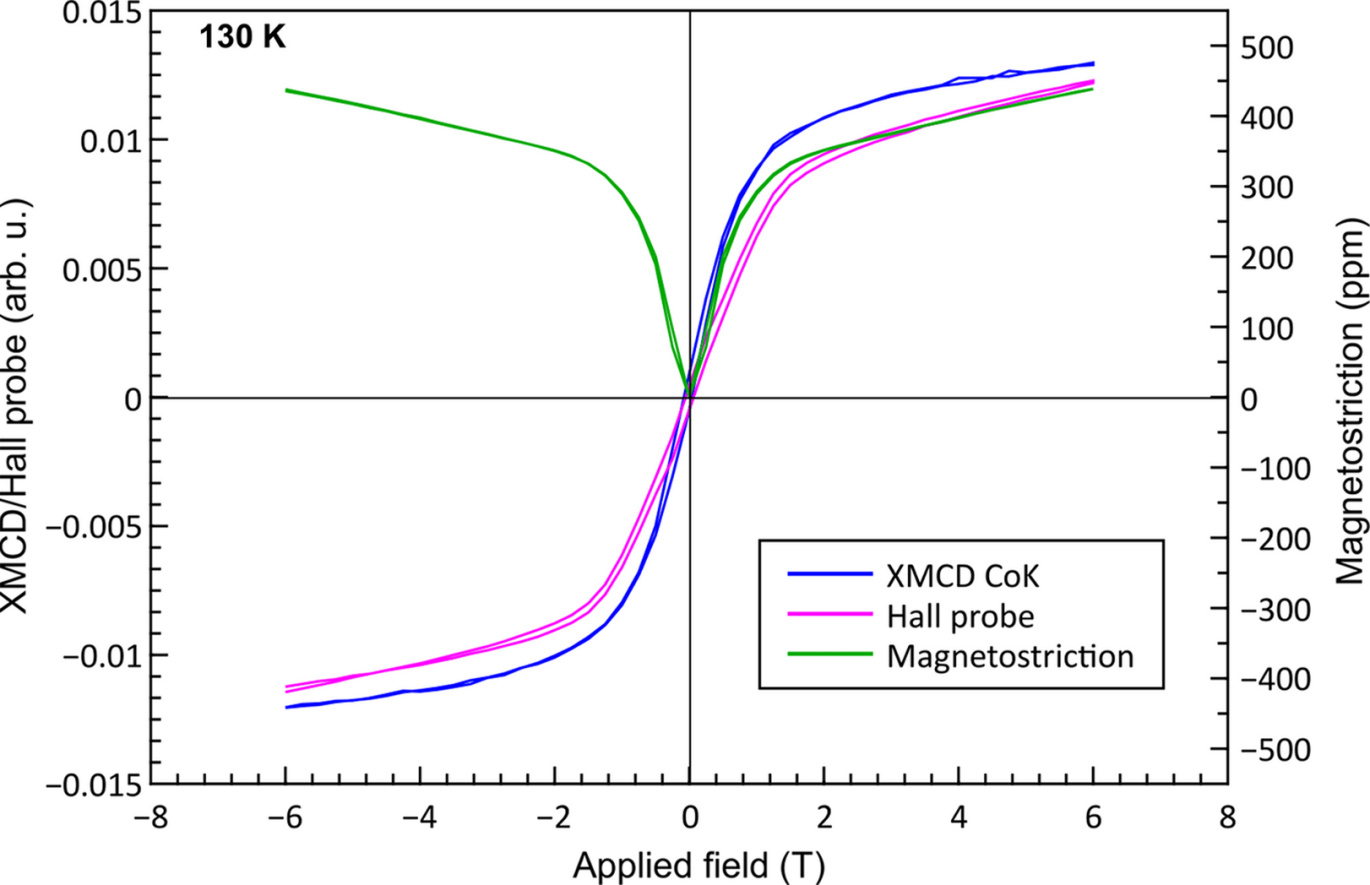
Hysteresis curve of XMCD (Co *K*-edge), magnetization and magnetostriction recorded simultaneously at 130 K.

In our case, XANES and XMCD experiments were performed at 10 K and 130 K under various applied magnetic fields up to 6.5 T (Fig. 7 ▸). At the Co *K*-edge, the XMCD spectral shape is very much different from a single broad negative peak observed in metallic Co (not shown). It exhibits a three-peak structure: one negative in the middle of the positive ones. This implies that the *R* 4*f* magnetic states strongly affect the Co 4*p* electrons and consequently the influence is superimposed on the *K*-edge spectrum (Rueff *et al.*, 1998 ▸; Boada *et al.*, 2010 ▸). Note that the XMCD spectral shapes are virtually identical at both 10 K and 130 K. The amplitude of the XMCD signal at 10 K is found to be about 25% more intense than at 130 K. The magnetic properties of Dy were also investigated by XMCD at the *L*_2_-edge under the same conditions. A small difference in the XMCD spectra recorded at 10 K and 130 K is observed at photon energies around 8585.7 eV that can be attributed to quadrupolar 2*p* → 4*f * transitions (Lill *et al.*, 2025 ▸). One can also notice that the intensity of the XMCD signal at low temperature is also increased by a factor 1.25. The capacity of our ULMAG device to simultaneously measure the hysteresis loop of magnetization, magnetostriction and XMCD is exemplified in Fig. 8 ▸, where a clear correlation between each subsytem is observed.

**Figure 9:**
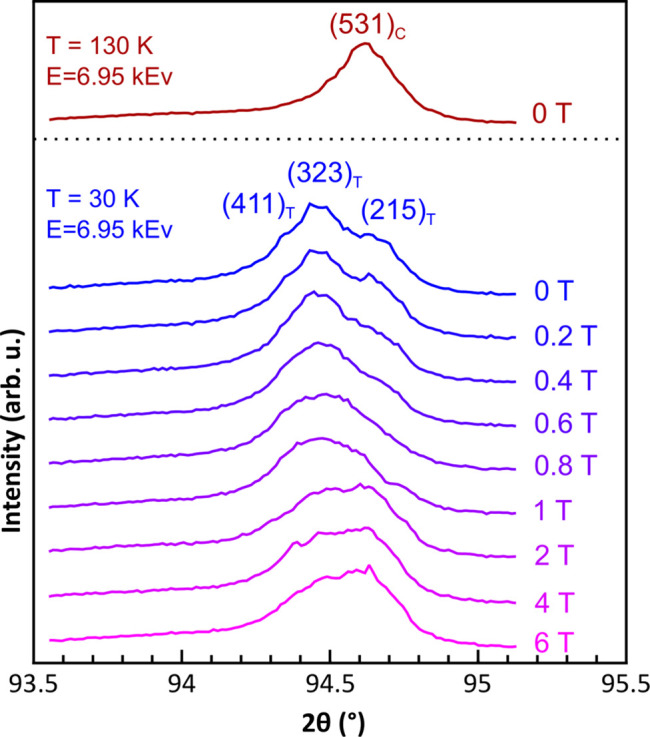
Evolution of the (531)_C_ diffraction peak of DyCo_2_ by decreasing the temperature from 130 K to 30 K and then increasing the applied magnetic field up to 6 T.

The cubic Laves phase structure (*Fd*3*m*, *a* ≃ 7.19 Å), present at *T* > *T*_c_, is distorted to tetragonal as the magnetic order set in (Yang & Ren, 2008 ▸; Nie *et al.*, 2013 ▸, 2017 ▸; Burzo *et al.*, 2017 ▸). The lattice distortion is very small (*c*/*a* ≃ 0.9986 at 120 K) and it is usually difficult to observe such a tetragonalization in laboratory devices, but detectable with high-resolution XRD measurements at synchrotrons (Yang & Ren, 2008 ▸; Nie *et al.*, 2013 ▸, 2017 ▸). The tetragonal structure can be described by the *I*4_1_/*amd* space group (*a* ≃ 5.08670 Å, *c* ≃ 7.17180 Å). It was shown that the large magnetostriction in this alloy was induced by tetragonal variants (Yang & Ren, 2008 ▸; Nie *et al.*, 2013 ▸, 2017 ▸; Burzo *et al.*, 2017 ▸). We investigated this tetragonalization using our ULMAG setup by monitoring the cubic (531) diffraction peak, measuring the XRD as a function of field. The photon energy was chosen to be below the *L*_2,3_-edges of Dy and the *K*-edge of Co (6.95 keV), which gives the (531)_C_ diffraction peak of the cubic high-temperature structure at 2θ = 94.54°. The results are shown in Fig. 9 ▸. A broad single peak is observed at 130 K, whereas it splits into three distinct peaks well below the Curie temperature (*i.e.* at 30 K), confirming the tetragonalization of the structure. The observed peaks are assigned as (411)_T_, (323)_T_ and (215)_T_ Miller indices. Furthermore, by applying a magnetic field, one observes the merging of the peak splitting together, suggesting a reorientation of tetragonal variants (Yang & Ren, 2008 ▸; Nie *et al.*, 2013 ▸, 2017 ▸). One should bear in mind that the XRD in reflection mode [see Fig. 3 ▸(*a*)] with the ULMAG instrument probes the diffracting plane orientated at nearly 45° with respect to the incident beam, whereas the magnetic field is applied parallel to it. Thus, a direct comparison with magnetostriction is only possible if the strain gauge is also orientated by 45° with respect to the field. Nevertheless, these results clearly show the ability of the setup to probe the structural transition induced by applied magnetic field or temperature with high resolution.

### 5.2. FeRh

Among the magnetocaloric materials with first-order transitions, FeRh alloys hold attention since the first published study by Fallot (1938 ▸). Across the antiferromagnetic (AFM) to ferromagnetic (FM) transition of FeRh, Δ*T*_ad_ = 12.9 K has been reported in a magnetic field of 1.95 T (Nikitin *et al.*, 1990 ▸), being one of the highest values ever recorded for any materials under a magnetic field change below 2 T. Even though bulk FeRh is not realistically suitable for application due to high content of the very critical Rh metal, it could actually find applications in thin film form (Thiele *et al.*, 2003 ▸; Fina *et al.*, 2020 ▸; Quintana *et al.*, 2023 ▸) and its bulk form is usually considered as a benchmark system for studying the first-order transition process. Both AFM and FM phases adopt a cubic CsCl-type structure (B2). A considerable lattice volume expansion (∼1%) is observed across the transition. In addition, the latter is accompanied by a large resistivity and magnetostriction changes, together with the magnetocaloric effect. Thus, it can serve as a prototypical system to study first-order phase transitions and the correlation between charge, spin and lattice degrees of freedom during the transition.

**Figure 10:**
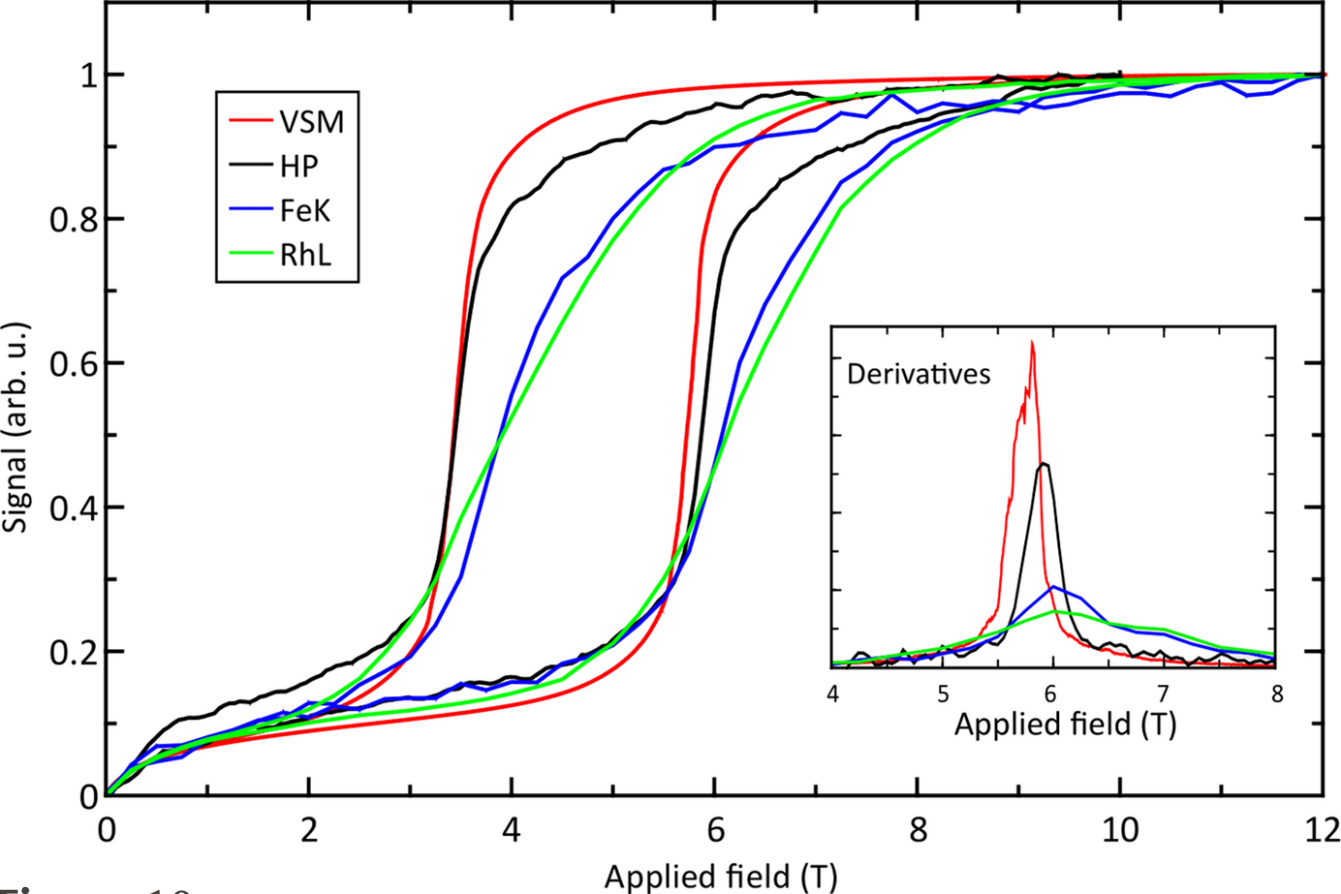
Comparison of the magnetic signal at 245 K as a function of magnetic field measured on the same FeRh sample with different techniques: VSM, HP, XMCD signal at the Fe *K*-edge and XMCD signal at the Rh *L*-edge. The inset presents the first derivative for each signal.

The element-selective magnetization curves (*i.e.* the intensity of the XMCD signal as a function of applied magnetic field either at the Fe *K*-edge or at the Rh *L*_2_-edge) were recorded together with the sample magnetization measured as a stray field using an HP (Aubert *et al.*, 2022 ▸). New measurements have been performed using the Asensor HPs, which provide much better signals. The corresponding results are plotted together with VSM data acquired at 245 K in Fig. 10 ▸. From the plot, one can observe that all signals saturate at an equivalent magnetic field (around 10 T), while a clear difference in the slope of the signal is observed during the transition depending on the technique used. This is highlighted in the inset, which shows the first derivative of each signal. Our first set of data (Aubert *et al.*, 2022 ▸) suggested that the difference between macroscopic measurement and XMCD could not be explained by an effect of the surface, since the XMCD curves are nearly identical for the Rh *L*_2_-edge and Fe *K*-edge, whereas the respective X-ray penetration depths differ by a factor of five (1 µm and 5 µm, respectively). However, after a deeper analysis, it is actually plausible that the observed difference is due to the surface/demagnetizing effect that leads to a difference between the macroscopic measurement (VSM, HP) and XMCD signal. One can see that the Rh *L*-edge (lowest penetration depth) shows the lowest slope, followed by the Fe *K*-edge, then the HP signal (which measured stray field near the sample surface) and finally the sharp transition is observed for the VSM signal (surface effect neglected). These measurements emphasize the need for careful consideration when comparing data obtained using different techniques, as they may pertain to either bulk or surface properties, which can differ.

**Figure 11:**
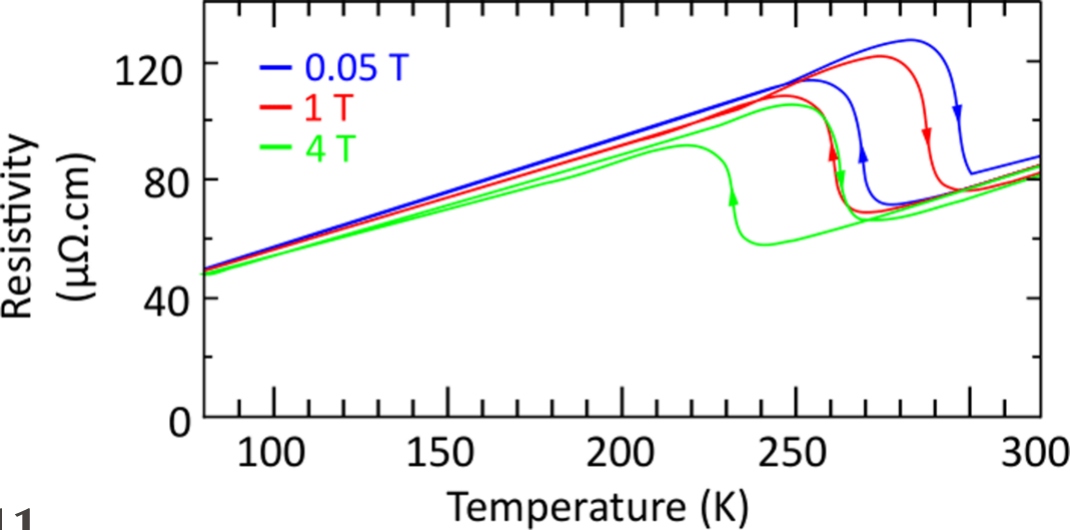
Resistivity signal of FeRh as a function of temperature for various magnetic fields.

In addition, the FeRh transition is accompanied by a large change in resistivity, as shown in previous studies. Here, a resistivity measurement was performed at ID12 as a function of temperature, showing the capability of our setup to measure the resistivity of metals (see Fig. 11 ▸). For various fields, the transition is clearly seen to shift temperatures, as the FM state is stabilized in a wider temperature range when a stronger magnetic field is applied (Aubert *et al.*, 2024 ▸). This option can be used to trace any changes in resistivity and correlate them with XAS/XMCD element-specific techniques.

## 6. Conclusions

This paper demonstrates the unique and versatile capabilities of the newly developed ULMAG instrument, which enables the measurements of various macroscopic properties (magnetization, volume change, resistivity, magnetocaloric effect *etc.*) concomitantly with measurements using X-rays such as element-specific XAS and XMCD, as well as XRD. Access to this instrument is available for any ESRF user upon request through the standard ESRF beam time application process.

The versatility of the instrument lies in the fact that each user can choose the specific properties to be measured based on their individual objectives and sample requirements, provided that the sensors are pre-mounted. This unique instrument opens new possibilities for exploring the intricate interplay among the magnetic, structural and electronic subsystems of functional materials across a wide range of applications.
